# Trehalose and maltodextrin preserve microbial community structure in freeze-dried fecal samples for fecal microbiota transplantation

**DOI:** 10.1093/ismeco/ycaf204

**Published:** 2025-12-12

**Authors:** Paul Oladele, Timothy A Johnson

**Affiliations:** Department of Animal Sciences, Purdue University West Lafayette, Indiana, 47907, United States; Department of Animal Sciences, Purdue University West Lafayette, Indiana, 47907, United States

**Keywords:** fecal microbiome, fecal microbiota transplantation, lyophilization, viable microbial community, bacteria, propidium monoaxide

## Abstract

Fecal microbiota transplantation (FMT) is a promising approach for restoring gut microbial balance in both humans and animals. However, the logistical limitations of transplanting fresh fecal samples have increased interest in freeze-dried (lyophilized) fecal material as a transplant inoculum. While lyophilization facilitates storage, it can compromise bacterial viability, which is essential for FMT effectiveness. Lyoprotectants are often used to protect bacterial cultures during freeze-drying, but their effect on complex microbial communities remains unclear, as they may preferentially preserve some taxa over others. This study investigated the impact of four lyoprotectants—mannitol, maltodextrin, trehalose, and a maltodextrin-trehalose mixture—on bacterial viability and community structure in pig fecal samples post-lyophilization. Propidium monoazide (PMA) treatment combined with 16S rRNA sequencing (PMAseq) was used to differentiate viable from non-viable bacteria. In the total community (without PMA), microbial profiles appeared similar across treatment groups. However, when focusing on the viable community (PMA-treated), lyoprotectant choice significantly influenced the post-lyophilization community composition. Gram-negative bacterial viability was especially sensitive to lyophilization. Trehalose and maltodextrin preserved bacterial viability and community structure more effectively than mannitol. Mannitol-treated samples had reduced viable bacterial cells and altered community composition, while trehalose and maltodextrin better maintained diversity and structure of the viable (PMA-treated) communities. Taken together, lyoprotectants have differential effects on microbial composition during lyophilization. Among those tested, trehalose and maltodextrin best preserved both viability and community structure, making them promising candidates for FMT applications. Future research should explore optimizing lyoprotectant formulations to enhance microbiome stability and functional outcomes.

## Graphical Abstract

**Graphical Abstract:**
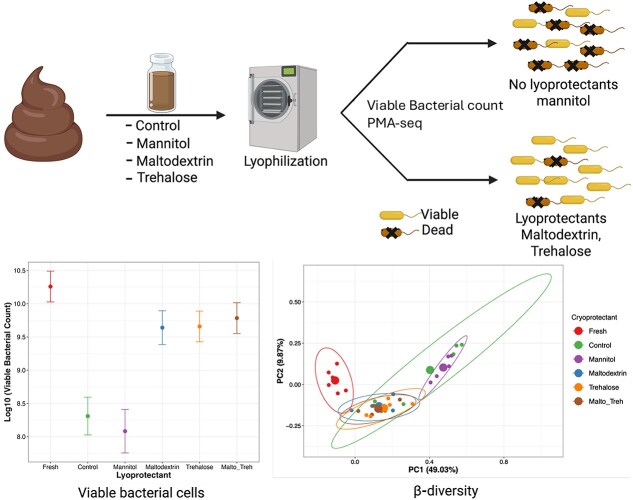

## Introduction

Fecal microbiota transplantation (FMT) is emerging as an important therapeutic option for both animal and human health. In humans, FMT is highly effective in treating *Clostridioides difficile* infection [1] and has been used experimentally for neurodegenerative diseases [2], inflammatory bowel disease [3] and other gastrointestinal tract infections [4]. In animals, FMT has been experimentally used for gastrointestinal tract infections like enterotoxigenic *Escherichia coli* infection during the weaning period in piglets [5, 6]. Because FMT involves transferring an intact microbial community from a healthy donor to a recipient, initial efforts have depended on using fresh fecal inoculum as the transplant material [7]. However, the use of fresh inoculum is logistically challenging. Comprehensive pathogen screening is also difficult to achieve with fresh fecal matter because some tests require hours, and the same fecal inoculum could not be used for different recipients over an extended period of time [8].

Due to these challenges, researchers have begun to use standardized frozen or freeze-dried fecal preparations for FMT [8–10]. The freeze-drying process can damage bacterial cells, reducing the number of viable cells for subsequent FMT [11]. Bacterial viability is generally assumed to be critical for FMT clinical efficacy, since the efficacy of FMT is thought to be through the colonization of these bacterial cells in the intestinal tract of the recipient [12]. To preserve bacterial cell integrity during freeze-drying, there is a need for the use of materials that protect bacterial cells during the freezing process [13], either through the displacement of water to stabilize the phospholipid bilayer or by preventing the formation of ice crystals during freezing [14]. Glycerol, which is usually used as a cryoprotectant, cannot be used as a lyoprotectant because its viscosity leads to a sticky end-product, which is not amenable for subsequent FMT [13]. Lyoprotectants like sucrose, mannitol, maltodextrin, and trehalose have been shown to produce powder-like end-products and are effective in reducing bacterial cell damage caused by freeze-drying [15], and subsequently improve the effectiveness of FMT [13, 16]. A previous study by Staley *et al.* reported that fecal inoculum treated with trehalose had higher number of viable bacterial cells compared to mannitol treated samples after freeze-drying [13]. The number of viable cells observed in samples preserved with trehalose was comparable to those preserved with glycerol prior to freezing. Sedeek *et al.* (2025) reported that trehalose preserved the microbial community structure of human fecal microbiota after lyophilization [17]. However, the efficacy of other lyoprotectants in maintaining bacterial community structure after lyophilization is unclear.

Cell viability in the microbial community is critical to the outcomes of FMT, but DNA-based microbial metagenomics sequencing methods do not differentiate between viable and non-viable bacteria cells. Treatment of bacterial cells with propidium monoazide (PMA), a DNA intercalating dye, can be used to distinguish between viable and non-viable members of the community when coupled with a sequencing-based approach of profiling microbial communities [18, 19]. PMA cannot penetrate intact and viable cells, but PMA can penetrate and bind DNA of damaged bacterial cells after photoactivation. The combination of PMA treatment and 16S rRNA sequencing (PMA-seq) has been shown to effectively differentiate between viable and dead bacteria in fecal microbial community [20].

Although, no studies have specifically examined the effect of lyoprotectants on the structure of fecal microbial communities after freeze-drying, there are reports of cryoprotectants having a significant effect on the structure of fecal microbial community after freezing largely due to differences in their ability to effectively preserve individual genera [21, 22]. We expect lyoprotectants to have similar effect on fecal microbial community after freeze-drying. Specifically, we hypothesized that while lyoprotectants would preserve bacterial viability, they would differentially maintain the community structure of the viable portion of pig fecal microbiota. To test this hypothesis, we treated fresh pig fecal communities with one of four lyoprotectants (mannitol, maltodextrin, trehalose, or a combination of maltodextrin and trehalose) and then determined bacterial viability and microbiome structure after lyophilization. In this study, we used a monosaccharide (mannitol), disaccharide (trehalose), and polysaccharide (maltodextrin) because the different number of sugar molecules they contain could impact their ability to protect bacterial cells from damage during freeze-drying.

## Materials and methods

### Sample collection, DNA extraction, and library preparation

Fresh fecal samples were collected from six 12-week-old piglets by rectal palpation at the Animal Science Research and Education Center at Purdue University according to protocol 2 108 002 177 approved by the Purdue University Institutional Animal Care and Use Committee (IACUC). Within 30 min of collection, fecal samples were transported on ice from the farm to the laboratory. Fecal samples were homogenized before being diluted 1:1 in sterile lyoprotectants reconstituted with phosphate buffered saline (PBS). Four lyoprotectants (5% mannitol (Fisher Scientific, Fair Lawn, NJ, USA), 5% maltodextrin, 5% trehalose, and a combination of 2.5% maltodextrin and trehalose) were used. The control group was treated with PBS rather than a lyoprotectant. The processed samples were then transferred to sterile jars (20.3 × 20.3 × 23.6 cm) (Ball Corporation, Danville, IN, USA) and frozen at −20°C before freeze-drying with the Labconco FreeZone Bulk Tray dryer (Marshall Scientific, Hampton, NH, USA) at −84°C condenser temperature and vacuum pressure of 0.04 mBar.

For total bacteria and viable bacteria analysis, fecal slurry was diluted (1:10, w/v) in PBS to a final volume of 1000 μL. For the staining, 10 μL of the diluted slurry was used. The number of total and viable cells was determined using Quantom total cell and viable staining kits which uses membrane-permeable fluorescence dyes that stain nucleic acids. The samples were loaded into M50 Cell Counting Slides and counted on the QUANTOM Tx Microbial Automated Cell Counter (Logos Biosystems, Anandale, VA, USA) following the manufacturer’s instructions. The following parameters were used on the cell counter: minimum fluorescent object size of 0.3 μm, maximum fluorescent object size of 20 μm, size gating of 0.3–20 μm, roundness of 50%, declustering level of 7, detection sensitivity of 4, autofocus for focusing method, and LED level of 5.

For DNA extraction, samples were collected from the control samples immediately after processing and before freezing to serve as fresh sample control for all treatment groups. Samples were collected from all treatment groups after freeze-drying for DNA extraction. Another set of samples were collected (both fresh and freeze-dried) and subjected to PMA treatment before DNA extraction. For PMA treatment, 1 ml of PBS was added to 1 g of lyophilized sample and mixed well. Fresh samples were already suspended in PBS and additional PBS was not added. A 200 μl aliquot of the slurry was further mixed with 200 μl PBS and then mixed with 1 μl of 20 mM PMAxx™ (Biotium, Fremont, CA, USA) to make a final concentration of 50 μM in a 1.5 ml tube. The mix was incubated for 30 min at room temperature in the dark. After incubation, the sample mix was exposed to high density blue/white light (TruBlu 2 EDVOTEK, Washington, DC, USA) for 30 min. The tubes were placed horizontally and flipped after 15 min to allow light penetration into the samples. The photoactivated samples were centrifuged at 5000 g for 10 min to obtain pellets that were used for DNA extraction. Lyophilized samples not treated with PMA were added directly to DNA extraction tubes as a dry powder.

Total DNA was extracted from both PMA-free and PMA-treated samples using the PowerFecal Pro DNA kit (Qiagen, Germantown, MD) following the manufacturer’s instructions. The V4 region of the 16S rRNA gene was amplified, and a pooled amplicon library was prepared following the protocol described by Kozich *et al.* [23]. A mock community (20-Strain Even Mix 138 Genomic Material; ATCC® MSA-1002™) and water were used as controls during library preparation. The amplicon product for each sample was normalized with the SequalPrep Normalization Plate kit (Thermo Fisher Scientific, Frederick, MD, USA). The amplicons were then sequenced with an Illumina MiSeq Sequencer (2 × 250 paired end) at the Purdue University Genomics Core Facility.

### Sequence processing

Raw sequences obtained from 16S rRNA sequencing were analysed using Quantitative Insight into Microbial Ecology (QIIME2 v. 2022.8) [24] as previously described by Oladele *et al.* [25]. The raw sequences from the mock community were compared to the negative control (PCR grade water) to determine the presence of contaminants (Fig. S1). No contaminant ASV was discovered. The mock community had an error rate of 0.05% and reflected the expected composition. The water negative control sample only had 5336 sequences and was dominated by *E. coli* which is typical for our lab. Raw sequences were demultiplexed, and low-quality reads were removed during denoising with DADA2. During denoising, the forward and reverse sequences were trimmed at position 13, while they were truncated at positions 250 to obtain sequences with a 50^th^ percentile quality score > 35. Prior to rarefaction, the dataset contained an average of 26 528 reads per sample (SD = 11 152). Merged sequences were rarefied to a sampling depth of 13 924 reads per sample a threshold determined from the asymptote of the rarefaction curve, thereby normalizing sequence depth across all samples. After rarefaction, one sample from the viable community in the control group was lost and a total of 988 604 sequences were retained. Amplicon sequence variants (ASVs) were aligned with mafft, which was then used to construct a phylogeny with fasttree2. Alpha diversity metrics were estimated with Observed ASVs, Pielou’s evenness [26], and Faith phylogenetic diversity [27]. For beta diversity, the Bray–Curtis dissimilarity metric was used as estimates [28] and plotted as PCoA with R v. 4.2.2 [29]. Taxonomy was assigned to ASVs using a classifier trained with the V4 region (515F/806) of the Silva database (version 138) [30]. The database was prepared using the feature-classifier plugin in QIIME2, which extracts the target V4 sequences from the full-length 16S rRNA reference sequences and trains a naïve Bayes classifier for downstream taxonomic classification.

### Statistical analysis

Log transformed bacterial cells count data and alpha diversity metrics were analysed with a one-way ANOVA to test the difference between treatment groups at each time point. Tukey’s HSD test was used to compare multiple mean when ANOVA test was significant. Permutational multivariate analysis of variance test (PERMANOVA) in QIIME2 was used to test statistical differences in Beta diversity metrics (Bray–Curtis Dissimilarity Index) between treatment groups at each time point. Differential abundance analysis was conducted at the genus level with the QIIME2 implementation of ANCOM-BC, which models taxon-specific log-fold changes while correcting for compositional bias and unequal library sizes. Taxa present in fewer than 10% of samples were excluded using the default prevalence filter. The default Holm correction of False Discovery Rate in QIIME2 was used [31] and the output was imported into R for illustration. Significance threshold of 0.05 was set for ANCOM-BC. All figures were made in R v. 4.2.2 [29].

## Results

### Maltodextrin and trehalose preserved bacterial viability after freeze drying

**Figure 1:**
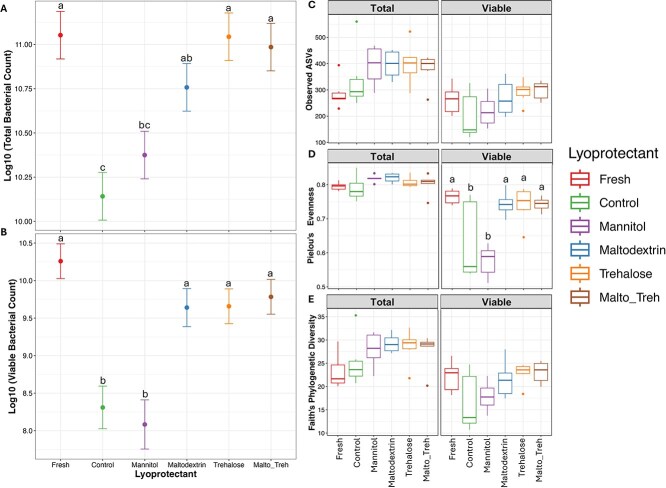
Effect of lyoprotectant on (A) Total bacterial count, (B) viable bacterial count, (C) observed number of ASVs, (D) Pielou evenness and, (E) Faith’s phylogenetic diversity. Panels A-B are derived from the Quantom assay data, while panels C-E are derived from sequencing data. Different superscripts indicate significant difference between treatment groups at *P* < .05.

The primary aim of using lyoprotectants prior to freeze-drying is to prevent bacterial cell damage and retain bacterial viability. We counted the number of total and viable bacterial cells with Quantum staining kits. As expected, the total number of bacterial cells decreased (*P <* .001) in the control samples after freeze-drying compared to fresh samples (Fig. 1A). There was also a decrease in the total number of bacterial cells in the mannitol samples (*P* < .001) after freeze-drying compared to fresh samples (Fig. 1A). However, there was no difference in the number of viable cells between the maltodextrin, trehalose, and maltodextrin-trehalose mixture and the fresh samples (Fig. 1B).

### Successful preservation of viable bacterial community structure by disaccharide and polysaccharide

The effect of lyoprotectant treatment on community structure was estimated by alpha and beta diversity. Three metrics were used for alpha diversity: Observed ASVs, Pielou’s evenness and Faith’s phylogenetic diversity. In all alpha diversity metrics, there was no significant difference between the treatment groups and the fresh sample in the total bacterial community (Figs 1C–E). However, in the PMA-treated (viable) samples, evenness was significantly lower (*P* < .001) in the lyophilized control and mannitol treated samples compared to fresh samples, while there was no difference between the fresh sample and the lyophilized samples treated with maltodextrin, trehalose, or the combination (Fig. 1D). Although there was no statistical significance, there was a similar trend of numerical decrease in number of observed ASVs and faith’s phylogenetic diversity in the lyophilized control and mannitol treated samples compared to the fresh samples in the PMA-treated samples (Fig. 1C and E).

**Figure 2:**
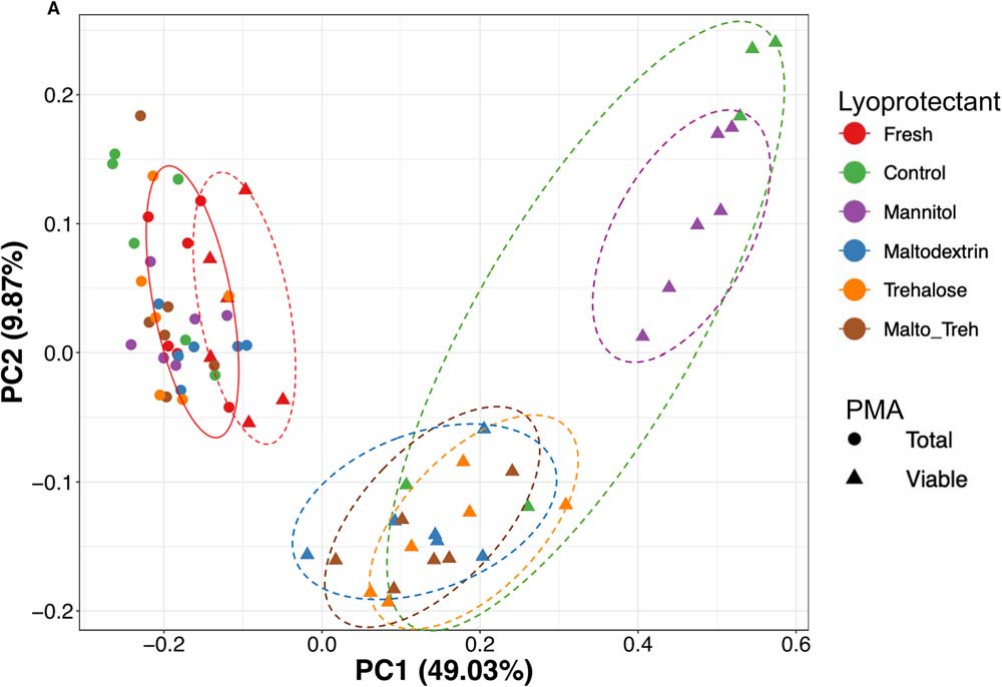
Effect of lyoprotectant on beta diversity estimated by Bray–Curtis Dissimilarity on both total and PMA-treated (viable) microbial community. The solid line encircles the total community, and broken line encircles the viable community in fresh fecal sample.

Community structure (beta diversity), estimated by Bray–Curtis dissimilarity index [28], indicated that both the total and PMA-treated (viable) components of the fresh sample displayed similar clustering patterns, indicating that PMA treatment did not cause significant change to the fresh microbial community (Fig. 2), which is consistent with the expectation that the majority of the cells were viable and therefore impermeable to PMA. The comparable profiles between treated and untreated fresh samples further support the efficacy of PMA in selectively excluding DNA from non-viable cells. As expected, lyoprotectant treatment did not significantly alter the total bacterial community structure, as shown by the clustering of lyophilized samples from all treatment groups with the fresh sample (Fig. 2). However, when considering samples treated with PMA (viable portion of community), control samples and mannitol treated lyophilized samples clustered together and away from fresh samples while lyophilized samples treated with maltodextrin, trehalose, or the maltodextrin/trehalose mixture clustered together and closer to the fresh samples (PERMANOVA *P* < .001, R^2^ = 0.57, Fig. 2).

### Depletion of microbial taxa after freeze-drying in control and mannitol treated groups

**Figure 3:**
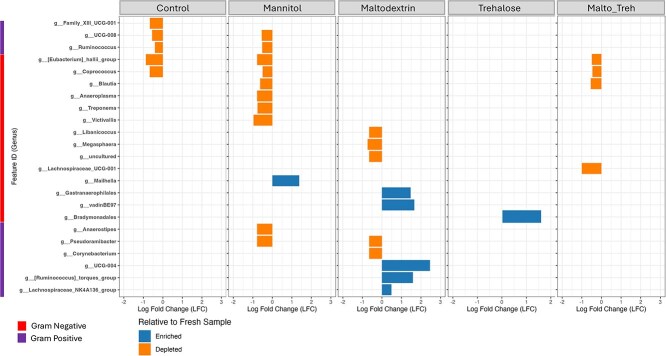
Differential abundant genera between the lyoprotectant treatment groups and fresh sample in the total community.

Based on the impact of the lyoprotectants on overall community structure, we determined a differential abundance of genera with ANalysis of COMpositions of microbiomes with Bias Correction (ANCOM-BC) which accounts for the compositional nature of microbiome data [31]. In the total community (not PMA-treated), there was minimal and inconsistent depletion or enrichment of genera in both gram-positive and gram-negative across all treatment groups when compared to fresh samples (Fig. 3). The highest depletion of taxa was observed in the mannitol treated samples with 10 genera depleted.

**Figure 4:**
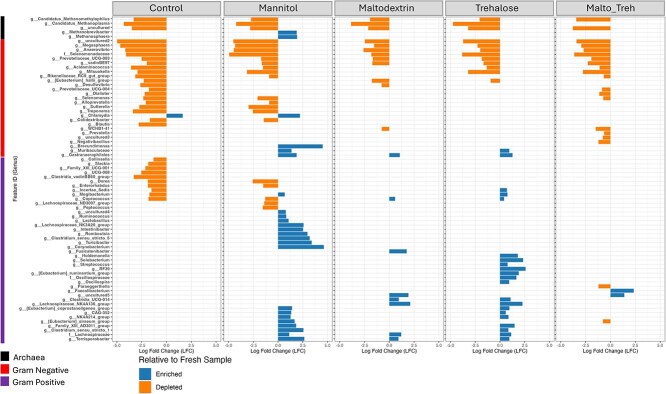
Differential abundant genera between the lyoprotectant treatment groups and fresh sample in the PMA-treated (viable) community.

However, in the PMA-treated samples (viable portion of community), the effect of freeze-drying and the protective effects of the lyoprotectants was more observable (Fig. 4). Freeze-drying had more effect on gram-negative taxa (19 genera depleted), while gram-positive and archaea had 10 and three genera depleted, respectively (Fig. 4). All 10 gram-positive genera depleted after freeze-drying were restored by maltodextrin, trehalose and their combination, but not in mannitol treated samples. None of the depleted archaea was restored by any of the lyoprotectants except in the maltodextrin-trehalose combination where *Candidatus Methanoplasma* was rescued. Of the 19 gram-negative genera depleted by freeze-drying, six genera—primarily from the orders Selenomonadales and Victivallales—were not rescued by any of the tested lyoprotectants. Five genera (*Blautia*, *Colidextribacter*, *Treponema*, *Sutterella,* and *Alloprevotella*) were rescued by both maltodextrin, trehalose or their combination. Maltodextrin rescued the highest number of taxa, rescuing six more taxa in addition to those rescued by maltodextrin/trehalose combination (*Selenomonas*, *Dialister*, Prevotellacaea_UCG_004, Rikenellaceae_RC9_gut_group, *Mitsuokella* and *Acidaminococcus*) while trehalose rescued five more taxa in addition to those rescued by maltodextrin/trehalose combination (*Selenomonas*, *Dialister*, Prevotellacaea_UCG_004, *Desulfovibrio*, and Rikenellaceae_RC9_gut_group).

In the PMA-treated samples, the control group had only 1 gram-negative taxa enriched (Fig. 4). In the lyoprotectant treatment groups, the combination of maltodextrin and trehalose had the least number of enriched taxa with two gram-positive genera enriched (Fig. 4). Mannitol treatment group had the highest number of enriched taxa, two archaea, six gram-negative and 18 gram-positive genera. Maltodextrin and trehalose had eight and 21 genera which are predominantly gram-positive. *Corynbacterium*, *Turicibacter*, *Clostridium* sensu stricto_6, *Romboutsia*, *Intestinibacter*, Lachnospiraceae NK3A20_group, *Lactobacillus*, *Ruminococcus* and *Peptococcus* were all enriched in mannitol treated group alone while *Oscillospira*, Bacilli_RF39, *Streptococcus*, *Solobacterium* and *Holdemanella* were all enriched only in trehalose treated group.

**Figure 5:**
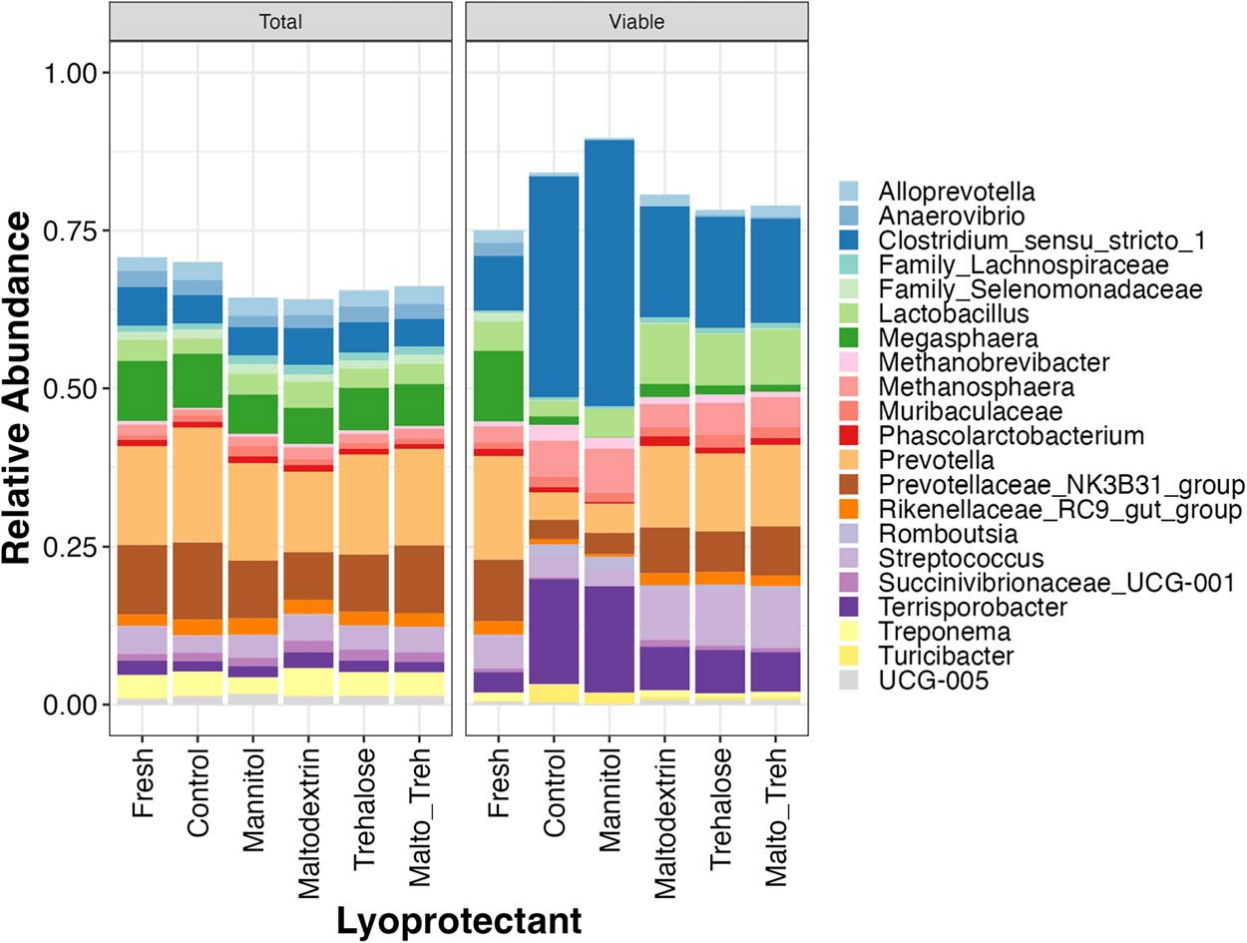
Taxa barplot of both total and PMA-treated (viable) microbial community.

Similar to result observed in the community structure, the relative abundance of taxa at genus level in the total community was similar among all treatment groups. However, in the PMA-treated samples, both control and mannitol treated samples had higher relative abundance of Clostridium_sensu_stricto_1 and *Terrisporobacter*. The relative abundance of *Lactobacillus*, *Prevotella*, Prevotellaceae_NK3B31_group and *Streptococcus* decreased in the control and mannitol treated samples (Fig. 5).

## Discussion

Recent studies have shown that lyoprotectants can effectively preserve over 50% of bacterial cells during lyophilization [13]. Freeze-dried fecal preparations have also shown efficacy comparable to fresh fecal inoculum in protecting against *C. difficile* infection in both mice and humans [9, 10, 32], as well as in preventing diarrhea in pigs when used for FMT [33–35]. However, the impact of different lyoprotectants on the microbial community structure remains unclear. In this study, we examined the effects of three commonly used lyoprotectants on the bacterial cell viability and microbial community structure in pig fecal inoculum prepared for FMT.

Our results show that lyophilization without lyoprotectant significantly reduced the number of viable bacterial cells and altered the viable bacterial community structure of the fecal samples. However, it should be noted that the staining procedure used for cell counting may not fully capture injured or metabolically compromised cells, which are neither completely permeable nor fully metabolically inactive, potentially leading to underestimation of partially damaged populations. Among the lyoprotectants tested, maltodextrin and trehalose ameliorated the negative effect of lyophilization, but mannitol did not. Interestingly, these protective effects were only observable in PMA treated samples, which represents the viable portion of the fecal microbial community. This suggests, as expected, that 16S rRNA amplicon sequencing amplifies DNA from viable and nonviable cells, and lyoprotectants successfully preserve a larger viable component of microbial community. This is critical to the efficacy of FMT, because only viable microbes can colonize the recipient animals.

The control treatment with PBS had depletion of numerous genera in PMA treated samples. A substantial proportion of these depleted genera were gram-negative bacteria, suggesting that the pronounced alteration in microbial community structure after freeze-drying may be primarily due to their loss. The vulnerability of gram-negative bacteria is multifactorial: in addition to having a thinner peptidoglycan layer compared to Gram-positive bacteria, they possess a complex outer membrane rich in lipopolysaccharides, which can be particularly sensitive to freeze-drying, osmotic stress, and desiccation [36, 37]. These structural features may reduce the effectiveness of mannitol’s protective mechanism. Mannitol functions by lodging between the bacterial cell wall and cell membrane, preventing the formation of ice crystals and thereby effectively reducing mechanical damage during the freeze-drying process [38]. Mannitol has also been reported to provide weak protection due to air bubble entrapment during the freeze-drying process [39]. Previous studies by Wang *et al.* and Staley *et al.* also indicated poor performance of mannitol as a lyoprotectant for *Lactiplantibacillus plantarum* and entire fecal microbial communities, respectively [13, 40], further reinforcing our observation of mannitol’s limited protective effect.

Maltodextrin and trehalose were effective in ameliorating the negative effect of freeze-drying, preserving both the number of viable bacterial cells and the microbial community structure. Both sugars effectively preserved all gram-positive bacteria depleted in the control and a greater proportion of gram-negative bacteria. Maltodextrin (a polysaccharide) and trehalose (a disaccharide commonly accumulated by plants as a response to dehydration [41]), share similar mechanism for preventing bacteria cell death during freeze-drying. They protect the cell membrane from damage by interacting with the lipid bilayer through hydrogen bonding [38, 42], and stabilize cells via glass formation through kinetic stabilization [43], with maltodextrin showing a more pronounced effect [44].

Interestingly, the combination of maltodextrin and trehalose was less effective compared to the use of each sugar individually. In our study, the mixture of maltodextrin and trehalose produced a similar effect on the number of viable bacteria and the microbial community structure as their individual treatment. However, there was a greater depletion of microbial taxa with the combination. Previous studies have suggested that combining disaccharides and polysaccharides can have a synergistic effect in improving viability of freeze-dried products [45]. Additionally, a maltodextrin-trehalose mixture has been reported to be optimal for freeze-drying fecal material for FMT [9, 46]. These studies primarily focused on the number of viable bacterial cells but did not consider the broader effects on the microbial community. Our findings highlight the need to assess not only bacterial cell viability but also the impact on the overall microbial community structure.

Mannitol and trehalose caused an increase in relative abundance of some bacterial taxa, primarily among gram-positive genera. Considering the compositional nature of 16S rRNA sequencing data, an increase in relative abundance could be due to an increase in absolute abundance or by maintaining absolute abundance and reducing the abundance of the rest of the community. An increase in absolute abundance is unlikely, since the fecal slurries were immediately frozen post-treatment, halting metabolic activity. Alternatively, an increase in relative abundance may simply indicate lyoprotection for specific bacterial taxa. In the case of mannitol e.g. *Corynebacterium,* which showed the highest enrichment in the mannitol-treated group, has been reported to possess a transporter for facilitating intracellular transport of mannitol [47]. This affinity may contribute to its enrichment; however, these findings highlight the nuanced interaction between bacteria and lyoprotectants, emphasizing the need for further investigation into how different sugars influence microbial community structure during preservation. When considering the magnitude of change in relative abundance, the largest changes (up to ±5 log fold change) occurred in the mannitol group and the smallest magnitude changes occurred in the maltodextrin group (mostly <2.5 log fold change). The magnitude of the changes also likely indicates the degree to which the community was altered, again indicating that maltodextrin was a preferred lyoprotectant.

In this study, we used PMA treatment to determine the effect of different lyoprotectant treatments on viable bacterial cells. While several studies have successfully used PMA to profile viable bacteria in mixed community, such as fecal, oral and environmental samples [18–20, 48, 49], other reports have shown important limitations in its quantitative accuracy in complex microbial communities where bacterial concentration and composition are unknown [50, 51]. In our study, PMA was therefore employed primarily to assess relative changes in the structure of the microbial community rather than to provide precise quantitative estimates of viability. To minimize potential bias, we compared the microbial community structure between total (untreated) and viable (PMA-treated) fractions in fresh fecal samples, which are expected to contain minimal numbers of dead bacterial cells. As anticipated, we observed no significant differences in beta diversity between the PMA-treated and untreated samples, supporting the assumption that fresh fecal samples predominantly consist of viable bacterial cells. On the other hand, when comparing the community from fresh samples to the control and mannitol-treated samples, which had large numbers of non-viable cells, we observed major shifts in the microbial community when we pre-treated the samples with PMA. These results indicate that samples pre-treated with PMA likely excluded non-viable cells from being amplified during library preparation, supporting the appropriateness of PMA for comparative, community-level analyses in this context.

In this study, we employed pig fecal samples to evaluate the impact of different lyoprotectants on microbial community viability. Pigs are widely recognized as a translational model for human gastrointestinal studies due to their physiological, anatomical, and dietary similarities, as well as overlap in core microbial functions [52]. However, important differences exist between pig and human gut microbiomes, particularly in the relative abundance of specific taxa and host-specific ecological interactions [52]. Such differences could influence the efficacy of lyoprotectants, as microbial survival during freeze-drying and reconstitution may depend not only on bacterial physiology but also on the broader ecological context in which taxa reside. Therefore, while our findings provide valuable insights into lyoprotectant performance, further validation in human-derived microbiomes will be essential to confirm their translational applicability.

Our study has some limitations. First, we evaluated the effect of lyoprotectants at a single time point immediately after lyophilization. Since the primary purpose of lyophilization is to enhance long-term storage, future studies assessing microbial viability and community structure over extended storage periods (1 or 2 years) would provide more comprehensive insights. A lyoprotectant only negative control would elucidate the concentration of contaminating bacteria from the lyoprotectant itself. Additionally, in future experiments, samples not treated with PMA should also be suspended in PBS and centrifuged to mimic the PMA-treatment to remove any bias this procedure may introduce.

## Conclusion

In summary, this study demonstrates that the choice of lyoprotectant significantly affects the preservation of fecal microbiota during freeze-drying. Trehalose and maltodextrin proved effective in maintaining bacterial viability, diversity, and community structure, making them suitable candidates for preserving microbiome samples for research and therapeutic applications. The effect of different lyoprotectants on the bacterial community was only observable in the viable fraction of the community. Future work should focus on optimizing lyoprotectant formulations to further enhance the preservation of key microbial taxa and functions, ultimately improving the outcomes of microbiome-targeted interventions in livestock and human health.

## Supplementary Material

Cryo_SuppMaterial_11_03_2025_ycaf204

## Data Availability

All raw sequencing reads are available in the NCBI sequence read archive (SRA) under the project accession PRJNA1253677. All files and scripts used in data analysis for this study are available at https://github.com/oluwapaul/Fmt-Cryo.
