# Supplementary material for: Trehalose and maltodextrin preserve microbial community structure in freeze-dried fecal samples for fecal microbiota transplantation
Source: ISME Commun. 2025 Dec 12;5(1):ycaf204. doi: 10.1093/ismeco/ycaf204 (PMC12716274; doi:10.1093/ismeco/ycaf204)
Supplement: Cryo_SuppMaterial_11_03_2025_ycaf204 [file cryo_suppmaterial_11_03_2025_ycaf204.docx]

**SUPPLEMENTARY MATERIAL**

**Trehalose and maltodextrin preserve microbial community structure in freeze-dried fecal samples for fecal microbiota transplantation**

Paul Oladele and Timothy A. Johnson*

Department of Animal Sciences, Purdue University, West Lafayette, Indiana, USA

*Correspondence:
Timothy A. Johnson
email: [john2185@purdue.edu](mailto:john2185@purdue.edu)

270 S Russell St, room 2020

West Lafayette, IN

Phone: 765-494-8019


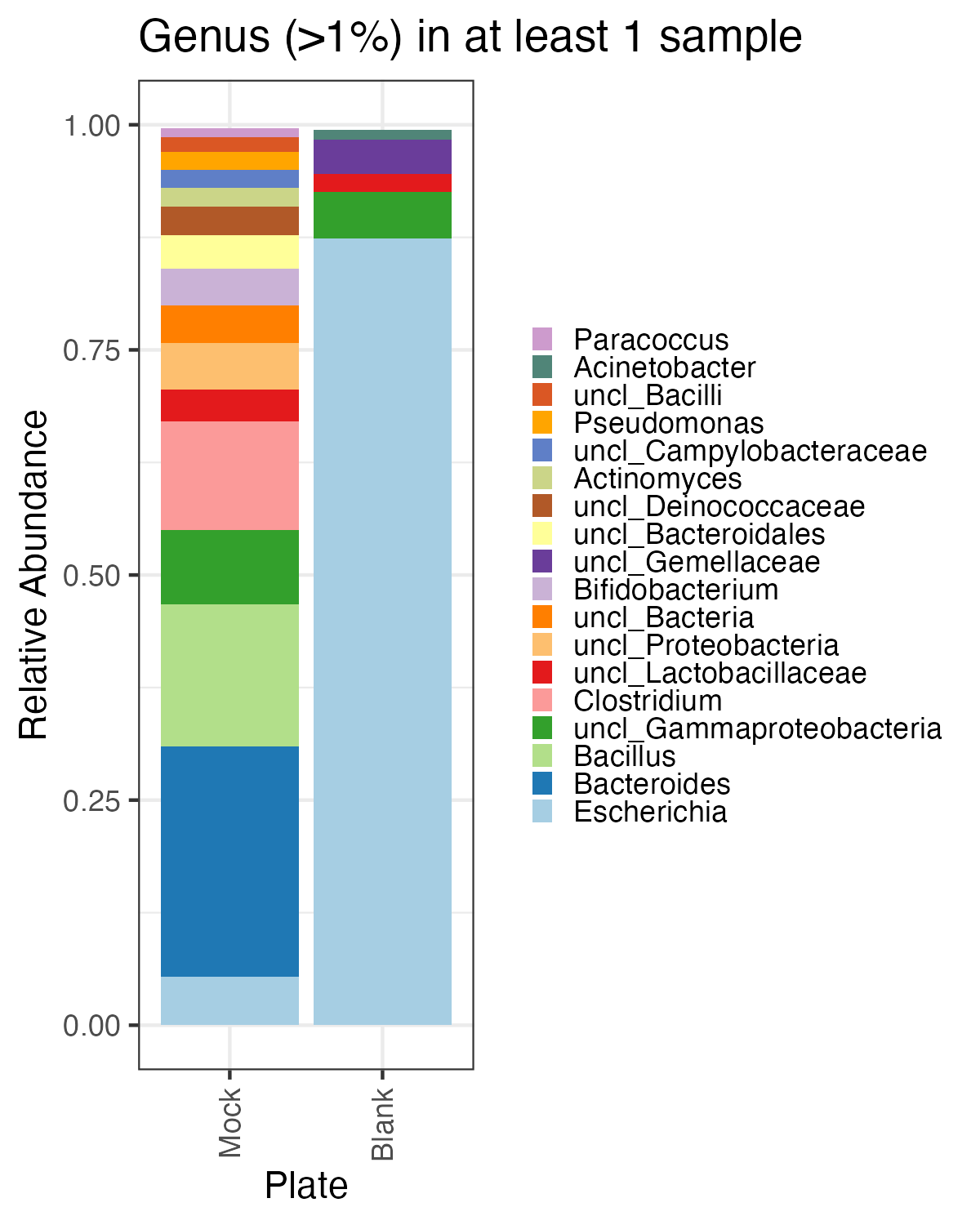


**FIG S1** Taxa barplot of both Mock and Blank (Nuclease free water) used as sequencing controls.
